# Comprehensive analysis of prognostic value and immune infiltration of kindlin family members in non-small cell lung cancer

**DOI:** 10.1186/s12920-021-00967-2

**Published:** 2021-05-02

**Authors:** Xiaoshan Su, Ning Liu, Weijing Wu, Zhixing Zhu, Yuan Xu, Feng He, Xinfu Chen, Yiming Zeng

**Affiliations:** 1 Department of Pulmonary and Critical Care Medicine, the Second Affiliated Hospital of Fujian Medical University, Respirology Medicine Centre of Fujian Province, Quanzhou, China; 2 Department of Thoracic Surgery, Fuzhou Pulmonary Hospital, Fuzhou, China; 3 Department of Pathology and Biomedical Science, University of Otago, Christchurch, New Zealand

**Keywords:** Kindlins, Non-small cell lung cancer, Bioinformatics, Prognosis

## Abstract

**Background:**

Kindlin Family Members have been reported to be aberrantly expressed in various human cancer types and involved in tumorigenesis, tumor progression, and chemoresistance. However, their roles in non-small cell lung cancer (NSCLC) remain poorly elucidated.

**Methods:**

We analyzed the prognostic value and immune infiltration of Kindlins in NSCLC through Oncomine, GEPIA, UALCAN, CCLE, Kaplan‑Meier plotter, cBioPortal, TIMER, GeneMANIA, STRING, and DAVID database. Additionally, the mRNA expression levels of Kindlins were verified in 30 paired NSCLC tissues and NSCLC cell lines by real-time PCR.

**Results:**

The expression level of FERMT1 was remarkably increased in NSCLC tissues and NSCLC cell lines, while FERMT2 and FERMT3 were reduced. Kindlins expressions were associated with individual cancer stages and nodal metastasis. We also found that higher expression level of FERMT1 was obviously correlated with worse overall survival (OS) in patients with NSCLC, while higher FERMT2 was strongly associated with better overall survival (OS) and first progression (FP). Additionally, the expression of FERMT2 and FERMT3 were obviously correlated with the immune infiltration of diverse immune cells. Functional enrichment analysis has shown that Kindlins may be significantly correlated with intracellular signal transduction, ATP binding and the PI3K-Akt signaling pathway in NSCLC.

**Conclusions:**

The research provides a new perspective on the distinct roles of Kindlins in NSCLC and likely has important implications for future novel biomarkers and therapeutic targets in NSCLC.

**Supplementary Information:**

The online version contains supplementary material available at 10.1186/s12920-021-00967-2.

## Introduction

Lung cancer is the most common cancer and the leading cause of cancer mortality, causing approximately 1,760,000 deaths worldwide each year [1]. Non-small cell lung cancer (NSCLC) accounts for approximately 80–85% of lung cancers, including lung adenocarcinoma (LUAD), lung squamous cell carcinoma (LUSC) and lung large cell carcinoma (LCC) [2]. Although gradual improvements in diagnosis and treatment therapies, the 5-year overall survival rate of NSCLC remains poor [3, 4]. Therefore, it is necessary to explore the mechanism underlying the tumorigenesis and progression of NSCLC and to search for novel biomarkers with high sensitivity and specificity.

The Kindlin family members are newly discovered focal adhesion proteins consisting of three members (FERMT1, FERMT2 and FERMT3) that share a conserved FERM domain-containing three subdomains (F1, F2 and F3) with an inserted pleckstrin homology domain. Remarkably, Kindlins may function as tumor promoters or suppressors depending on the cancer type. For instance, FERMT2 severs as a tumor-promoting role in gastric cancer [5], pancreatic cancer [6, 7] and breast cancer [8, 9], but as a tumor-suppressive role in epithelial ovarian cancer [10] and colorectal cancer [11]. Weinstein et al. reported a higher mRNA expression of FERMT1 in lung cancer tissues [12]. Besides, Zhan et al. also found that FERMT1 and FERMT2 had different expressions in lung cancer. FERMT1 was highly expressed in NSCLC, especially in LUSC. On the other hand, FERMT2 was highly expressed in LCC and weakly expressed in LUAD and LUSC [13]. Additionally, Djaafri et al. found that FERMT3 expression was decreased in lung cancer [14]. However, the series of research only reported the expression levels of Kindlins, and few studies have been conducted on the prognosis and mechanism of Kindlins.

Rapid advances in online platforms and various databases have contributed to the widely used bioinformatics analysis in the field of cancer research. Up to date, bioinformatics analysis has not been adequately used to investigate the roles of Kindlins in NSCLC. Hence, the present study investigated the role of individual kindlins in NSCLC using RT-PCR combined with bioinformatics. In this study, we aim to investigate the prognostic value and immune infiltration of Kindlins in NSCLC, providing new clues for early diagnosis, prognostic judgments and individualized treatments for NSCLC patients.

## Materials and methods

### Expression database of Kindlins in NSCLC

Oncomine database (http://www.oncomine.org) is a comprehensive cancer microarray database based on 86,733 samples from 715 databases for gene transcriptome analysis in different cancers [15]. The screening and data mining conditions included: “Gene: FERMT1, FERMT2 and FERMT3”; “Analysis Type: Cancer versus Normal Analysis”; “Cancer Type: Lung Cancer”; “Data Type: mRNA”; “*P* value < 0.05”; “Fold change:2”; “Gene rank: top 10%”. GEPIA database (http://gepia.cancer-pku.cn/) is a recently developed interactive online platform, applying for analyzing the RNA sequencing (RNA-Seq) expression based on over 9000 tumors from the TCGA and 8000 normal samples from the GTEx [16]. GEPIA was used to analyze Kindlins expression and tumor stage in LUAD and LUSC. UALCAN is a user-friendly web that provides publicly available cancer transcriptome data based on TCGA database [17]. UALCAN was used to confirm the association between mRNA expressions of Kindlins in NSCLC and clinicopathological parameters. CCLE (www.broadinstitute.org/ccle) provides the public information on gene expression, chromosome copy number and mutation profile of 947 human cancer cells [18]. In our research, we mainly use it to verify the expression levels of FERMT1, FERMT2 and FERMT3 in different kinds of cancer cell lines.

### Kaplan‑Meier plotter

Kaplan Meier plotter (http://kmplot.com/analysis) aims to discover and validate survival biomarkers based on a meta-analysis from 11 k samples from 20 different cancer types [19, 20]. In the study, we used the KM plotter database from a set of 1926 lung cancer samples to assess Kindlins’ prognostic values, including overall survival (OS), first progression (FP) and post-progression survival (PPS). Moreover, we evaluated the associations of the Kindlins with various clinical parameters of NSCLC, including the histology, clinical stages, gender and smoking history.

### cBioPortal

cBioPortal (http://cbioportal.org) is an open website used to explore, visualize and analyze multilayer cancer genome data from over 5,000 tumors from 20 different cancer studies [21, 22]. According to cBioPortal's online instructions, the Kindlins gene alterations information in different cancer types was obtained, including genetic mutations, gene fusions, gene amplifications, deep deletions and multiple alterations.

### TIMER

TIMER (https://cistrome.shinyapps.io/timer/) is a comprehensive database for analysis of the tumor-infiltrating immune cells from 32 cancer types [23]. In our research, Spearman correlation was used in the gene module to explore the correlation between the expression of Kindlins and immune infiltration, including tumor purity and six types of immune cells (B cells, CD8+ T cells, CD4+ T cells, macrophages, neutrophils, and dendritic cells).

### GeneMANIA and STRING database

GeneMANIA (http://www.genemania.org) is a useful and flexible prediction server, displaying a functional interaction network to explore the association between genes and data sets [24]. In the current study, we used GeneMANIA to analyze the relationships between Kindlins concerning the co-expression, co-localization, physical interactions, pathway, genetic interactions, prediction, and shared protein domains.

STRING (https://string-db.org/) is an online database, predicting protein–protein interactions network in terms of direct (physical) and indirect (functional) associations [25]. In this study, we constructed the protein–protein interactions network of Kindlins using and the selection criteria: “organism: Homo sapiens”; “the minimum required interaction score > 0.4”; “the max number of interactors: 20”.

### DAVID database

DAVID database (https://david.ncifcrf.gov) is a comprehensive bioinformatics enrichment platform, providing integrative and systematic annotations of biological functions from a series of genes/proteins [26, 27]. We applied the DAVID database (version 6.8) for gene ontology (GO) terms analysis and Kyoto Encyclopedia of Genes and Genomes (KEGG) pathways enrichment analysis of Kindlins and their related proteins. GO terms covers three aspects: biological processes, cellular components and molecular functions. The *P* Value < 0.05 was set as a criterion and regarded as significant enrichment.

### Lung tissue samples

In this study, between July 2019 and December 2019, thirty pairs of NSCLC tissue and adjacent normal tissues were collected from the Fuzhou Pulmonary Hospital, China. These tissues were used to detect the expression level of Kindlins mRNA by quantitative real-time PCR (RT-PCR). The collection and use of the samples were approved by the ethics committee of the Second Affiliated Hospital of Fujian Medical University. The approval number is 2019 (ethical research review)-207.

### Cell culture

The NSCLC cell lines (A549, SPCA-1 and H1299) and one normal cell line (BEAS‐2B) were obtained from Procell life science and Technology Co., Ltd (Wuhan, China). Human bronchial epithelial cells (BEAS-2B) were obtained from FenghBio Co., Ltd. (Changsha, China). All the cell lines were cultured at RPMI-1640 medium (GIBCO, Los Angeles, CA, USA) with 10% fetal bovine serum (FBS, Gibco) and grown in a humidified incubator at 37 °C (5% CO2) environment.

### RT-PCR analysis

Total RNA in tissues or cells was extracted using TRIzol reagent (Invitrogen) and cDNA was synthesized using Primescript RT Reagent (Takara Bio Inc., Japan). PCR amplification was performed using the SYBR Green PCR kit (Takara Bio Inc., Japan) in a 7500 PCR system (Thermo Fisher Scientific), and GAPDH was used as an endogenous control. The relative quantification analysis was performed using the comparative CT method. The following PCR primers were used:

- FERMT1 forward, 5′-TTGAAGATGGTGAGGTTGCGAGTC-3′
- FERMT1 reverse, 5′-GGGTTGGCTGAATGCGAGGATG-3′
- FERMT2 forward, 5′-TGGCTCTGGACGGGATAAGGATG-3′
- FERMT2 reverse, 5′-TTTGTGCTGAGGGGTGAACTGAAG-3′
- FERMT3 forward, 5′-ACTGCACCGAGGAGGAGATGATG-3′
- FERMT3 reverse, 5′-CCTTGAGGTTGAGCTGCTGAATGG-3′
- GAPDH forward, 5′-CTCCTGCACCACCAACTGCTTAG-3′
- GAPDH reverse, 5′-GACGCCTGCTTCACCACCTTC-3′

### Statistical analysis

RT-PCR was performed in triplicate. The data were analyzed using the GraphPad Prism (version 8.0). Student’s t-test was used to compare the expression of Kindlins mRNA between NSCLC tissue samples tissues and adjacent normal tissues. *P* < 0.05 was considered a statistically significant difference.

## Results

### Differential expression of Kindlins in NSCLC patients and cell lines

**Fig. 1:**
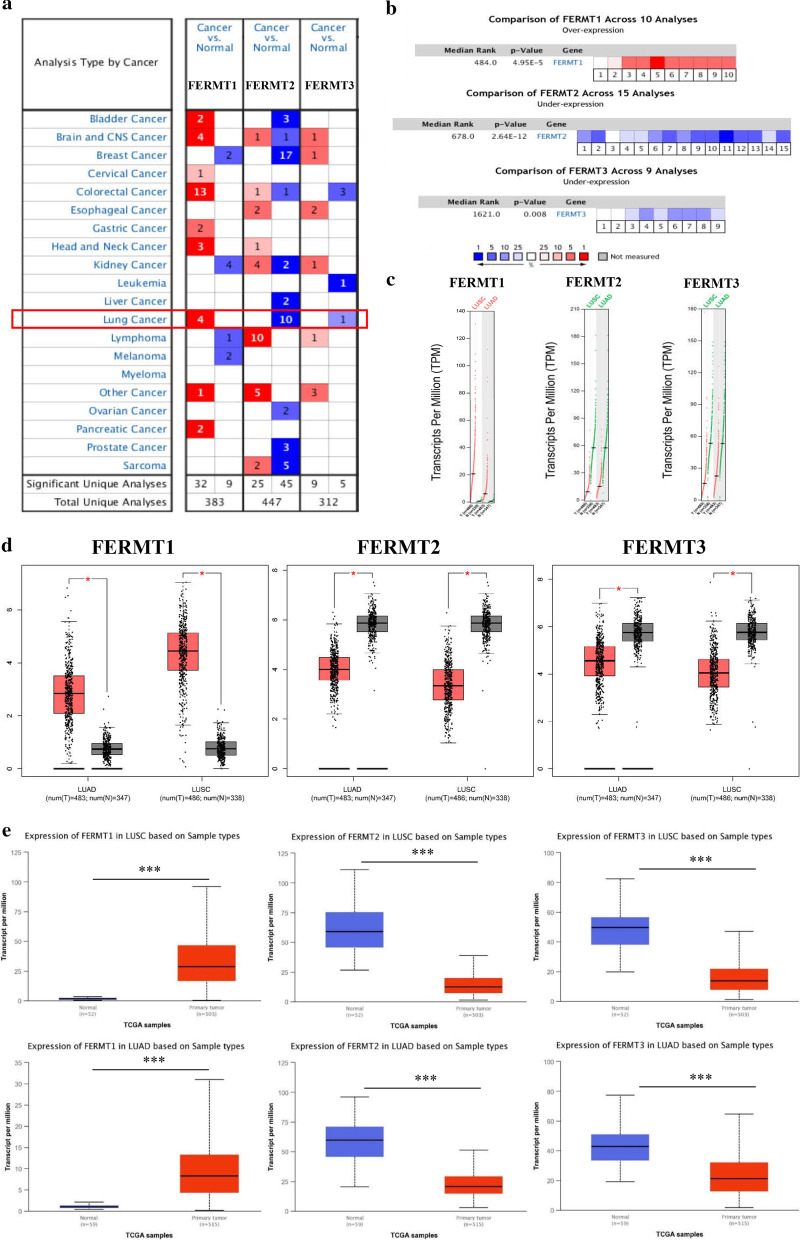
The expression of Kindlins in NSCLC. **a** The expression of Kindlins in different types of cancers compared with normal tissues (ONCOMINE). **b** Meta-analysis of FERMT1, FERMT2 and FERMT3 mRNA expression in NSCLC from multiple Oncomine databases. **c** The expression profile of Kindlins in LUAD and LUSD (GEPIA). Red trace, tumor samples; green trace, normal samples; T, tumor; N, normal. **d** The expression boxplots of Kindlins in LUSD and LUAD (GEPIA). A t-test was used to compare the expression level differences between tumor and normal tissues (*P* < 0.01). Y-axis represents log2 (TPM+ 1). Red box, tumor samples; black box, normal samples. T, tumor; N, normal. **e** The relative expression of Kindlins in LUAD and LUSD (UALCAN)

**Fig. 2:**
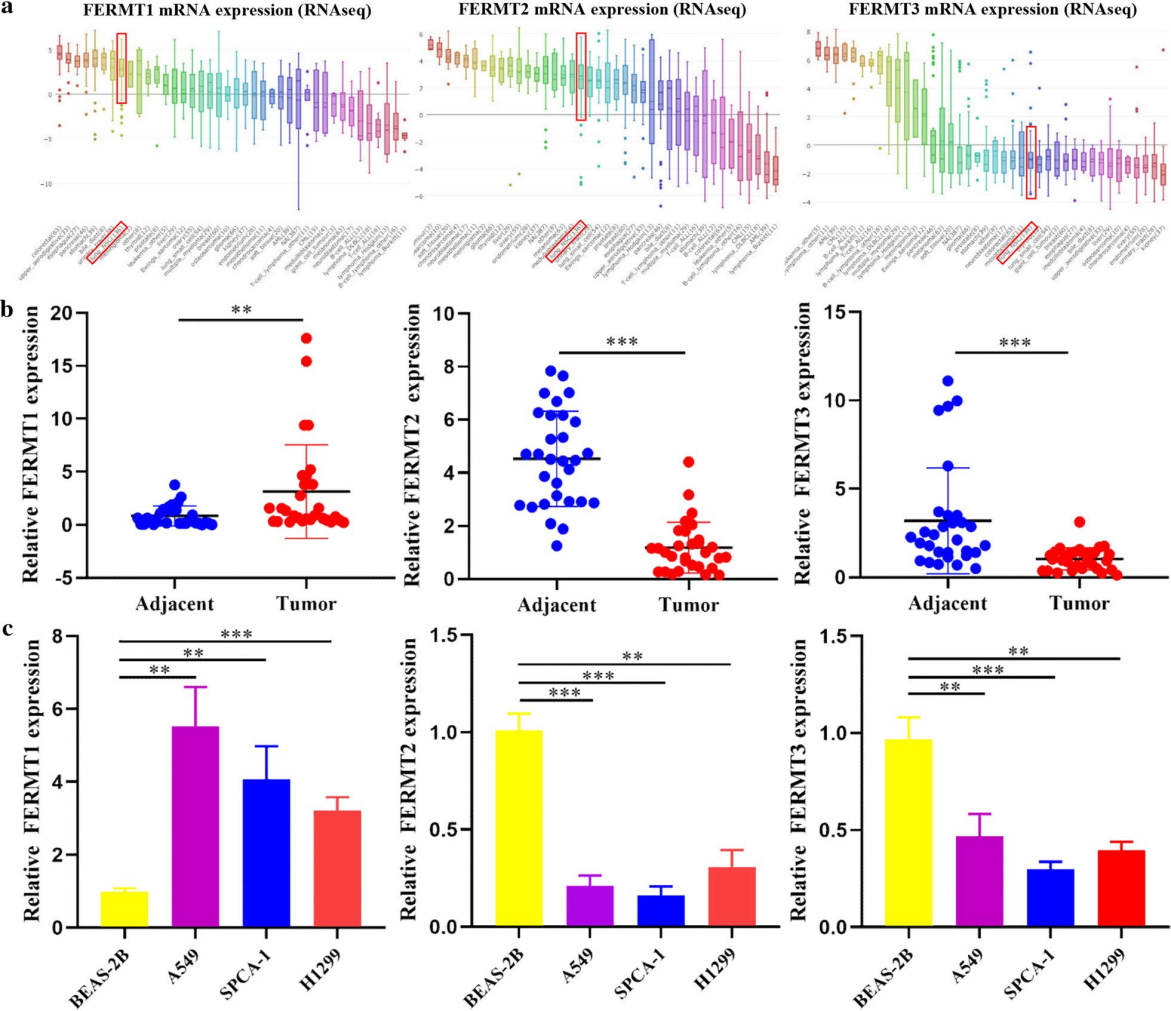
The expression of Kindlins in NSCLC tissue and cell lines. **a** FERMT1, FERMT2 and FERMT3 mRNA expression levels in NSCLC cell lines (CCLE). **b** Analysis of FERMT1, FERMT2 and FERMT3 expression in 30 pairs of NSCLC tissues and adjacent tissues in 41 NSCLC patients via RT-PCR assay. **c** Analysis of FERMT1, FERMT2 and FERMT3 expression in the NSCLC cell lines (A549, SPCA-1 and H1299) and one normal cell line (BEAS‐2B) via RT-PCR assay. **P < 0.05 ***P < 0.001

We first used ONCOMINE database to explore the expression of Kindlins in NSCLC. Multiple datasets showed that the mRNA expression level of FERMT1 was significantly increased in NSCLC tissues, while FERMT2 and FERMT3 were reduced in NSCLC versus normal tissues (Fig. 1a and Table 1,). These database include the Hou’s dataset [28], Su dataset [29], Okayama dataset [30], Wachi dataset [31], Selamat’s dataset [32], Landi’s dataset [33], and Stearman’s dataset [34]. Moreover, Meta-analysis of Kindlins genes expression in NSCLC studies from Oncomine databases was consistent with the above results (Fig. 1b). Then, we used the GEPIA and UALCAN dataset to further confirm these findings. The results indicated that the expression level of FERMT1 was overexpressed in LUAD and LUSC tissues than in normal tissues, while FERMT2 and FERMT3 were decreased inversely (Fig. 1c–e). We also used CCLE to explore expression levels of Kindlins in NSCLC cell lines. As presented in Fig. 2a, the expression level of FERMT1 was higher in NSCLC cell lines and FERMT3 was downregulated. These results were consistent with those from Oncomine, GEPIA and UALCAN dataset. Different from these results, FERMT2 expression was increased in NSCLC, which requires further examination.

**Table 1:** The significant changes of Kindlis in transcription level (ONCOMINE database)

	Comparison groups	Fold change	*P* value	t-test	Sample size	References
FERMT1	LUSC versus Normal	11.152	**1.76E−24**	23.513	92	[28]
	LUAD versus Normal	3.430	**1.57E−10**	7.780	110	[28]
	LUAD versus Normal	2.190	**1.26E−6**	5.426	57	[29]
	LUAD versus Normal	2.058	**1.39E−11**	9.585	246	[30]
	LUSC versus Normal	2.993	**2.20E−4**	7.925	10	[31]
FERMT2	LUAD versus Normal	− 2.029	**5.71E−13**	− 8.760	110	[28]
	LCC versus Normal	− 2.873	**6.81E−8**	− 7.776	84	[28]
	LUSC versus Normal	− 3.049	**1.22E−10**	− 9.147	94	[28]
	LUAD versus Normal	− 2.571	**5.08E−30**	− 15.496	116	[32]
	LUAD versus Normal	− 3.053	**9.39E−11**	− 8.536	57	[29]
	LUAD versus Normal	− 2.417	**6.56E−24**	− 13.005	107	[33]
	LUAD versus Normal	− 2.590	**3.44E−8**	− 8.11	39	[34]
	LUSC versus Normal	− 4.045	**1.21E−4**	− 6.837	10	[31]
FERMT3	LCC versus Normal	− 3.541	**8.33E−8**	− 7.916	84	[28]

LUAD, lung adenocarcinoma; LUSC, lung squamous cell carcinoma. LCC, lung large cell carcinoma. Significant* p*-value in bold (threshold* p* ≤ 0.05)

To verify the above results, we detect the mRNA expression level of Kindlins in 30 pairs of NSCLC tissues and NSCLC cell lines using RT-PCR. As expected, the results indicated that the FERMT1 expression was upregulated, while FERMT2 and FERMT3 were down-regulated in the tissue samples from 30 NSCLC cases (*P* < 0.05, Fig. 2b). Furthermore, the expression level of FERMT1 was highly expressed in NSCLC cell lines (A549, SPCA-1 and H1299) compared with normal cell lines (BEAS‐2B) (*P* < 0.05, Fig. 2c). For FERMT2 and FERMT3, the mRNA expression levels were reduced in NSCLC cell lines (*P* < 0.05, Fig. 2c). In conclusion, FERMT1 was significantly up-regulated in NSCLC patients and NSCLC cell lines, and FERMT2 and FERMT3 were significantly down-regulated.

### Association between the Kindlins expression and clinicopathological parameters in NSCLC

**Fig. 3:**
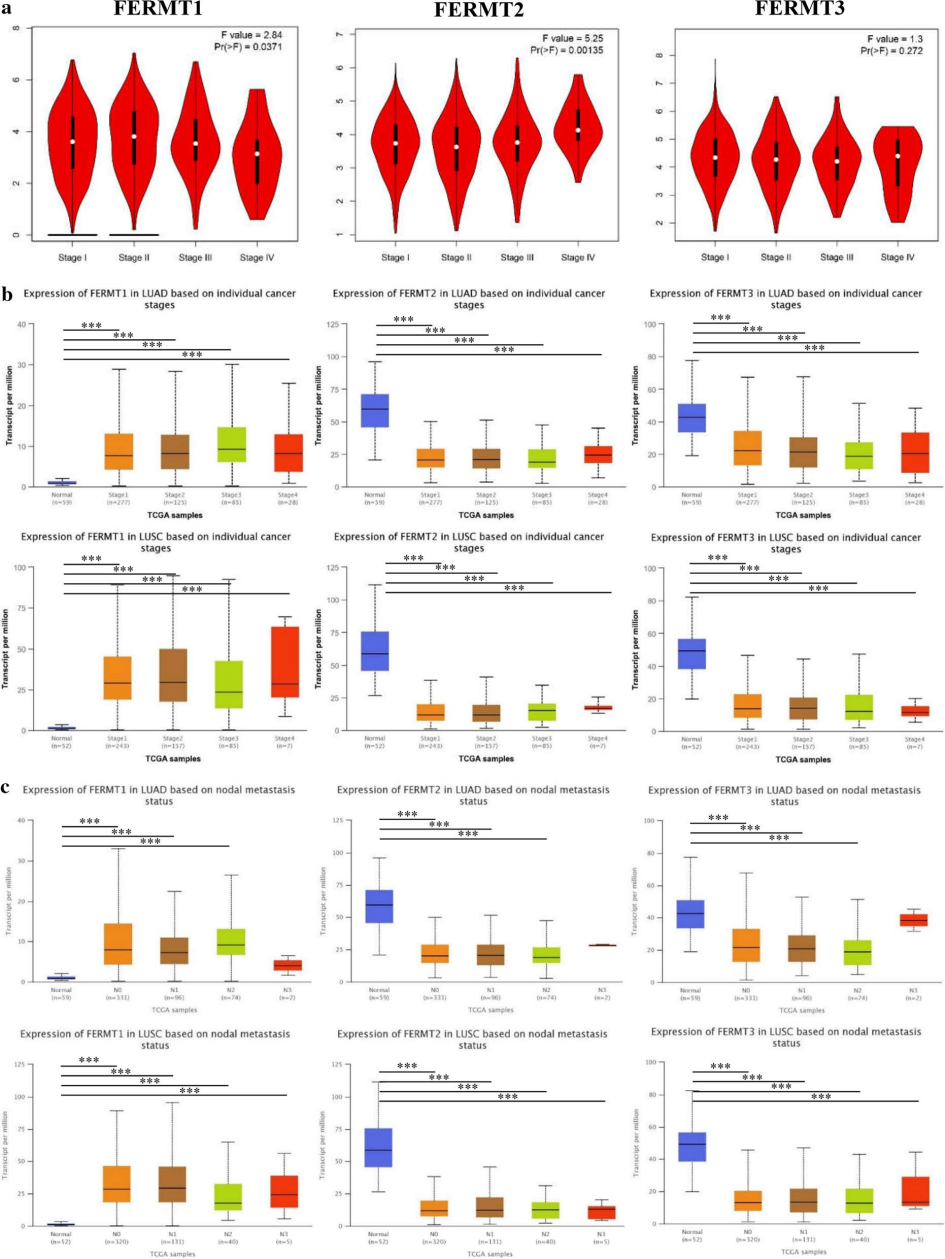
Association between the Kindlins expression and clinicopathological parameters in NSCLC. **a** Correlation between Kindlins expression and tumor stages in patients with NSCLC (GEPIA). **b** The correlations between Kindlins mRNA expression and clinicopathological stages in LUAD and LUSD (UALCAN). **c** The correlations between Kindlins mRNA expression and nodal metastasis status in LUAD and LUSD (UALCAN). *P < 0.05, **P < 0.01,***P < 0.001

We then further investigated the associations between the Kindlins mRNA expression and tumor stages in NSCLC using GEPIA and UALCAN datasets. As presented in Fig. 3a, the expression levels of FERMT1 and FERMT2 were remarkably distinct in different tumor stages of LUAD and LUSC, while there was no significance between FERMT3 and different tumor stages. We then explored the expression of Kindlins in distinct individual clinicopathological stages and nodal metastasis status of LUAD and LUSC using UALCAN dataset. It was shown that FERMT1 was highly expressed compared to normal tissues in every clinicopathological stage of LUAD and LUSC, while FERMT2 and FERMT3 genes were downregulated (Fig. 3b). Besides, the expression levels of Kindlins were significantly associated with nodal metastasis status (Fig. 3c). However, the expression levels of the Kindlin family members in different clinicopathological stages and nodal metastasis status were not different. These data suggested that FERMT1, FERMT2 and FERMT3 might play important parts in the tumorigenesis and progression of NSCLC.

### Prognostic analysis of Kindlins in patients with NSCLC

**Fig. 4:**
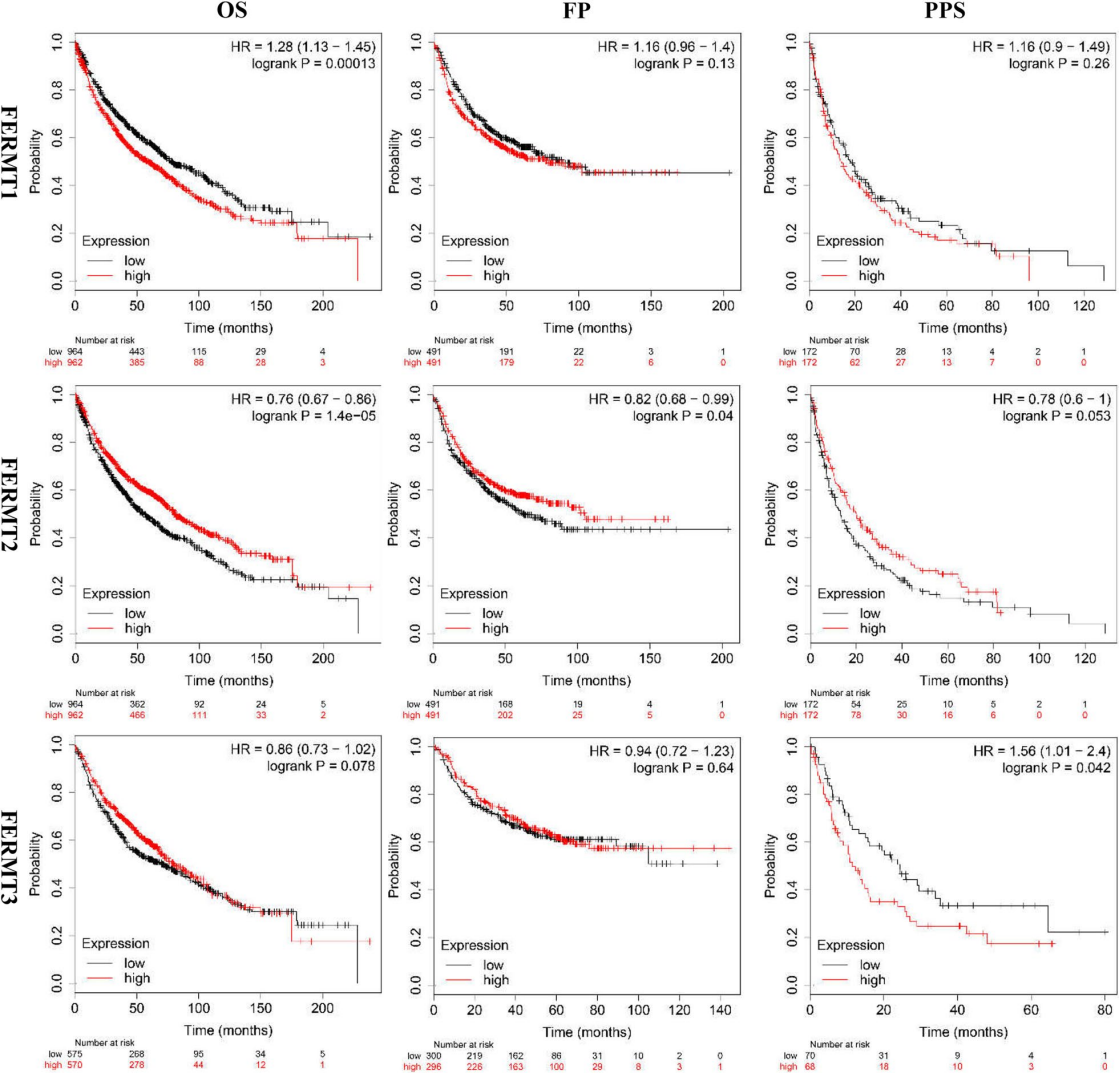
The prognostic value of the mRNA level of individual Kindlins in NSCLC patients (Kaplan–Meier plotter). OS, overall survival; FP, first progression; PPS, post-progression survival

Furthermore, we investigated the prognostic significance of Kindlins expression in NSCLC using Kaplan–Meier Plotter. We found that higher FERMT1 expression was significantly correlated with shorter OS in patients with NSCLC (HR = 1.28, log-rank *P* = 0.00013), while higher FERMT2 expression was strongly associated with better long-term OS (HR = 0.76, log-rank *P* = 1.4e−05) and FP (HR = 0.82, log-rank *P* = 0.04) (Fig. 4). Besides, patients with high FERMT3 mRNA levels had longer OS, but the difference was not significant (HR = 0.86, log-rank *P* = 0.078). Moreover, the expression of FERMT2 was correlated with favorable OS in NSCLC patients with stage 1, suggesting that FERMT2 might act as a prognostic role in early-stage NSCLC (S1). Taken together, the findings point out that FERMT1 expression might be a danger factor, whereas FERMT2 expression might be a protective factor for the prognosis in NSCLC.

### Alterations of Kindlins in patients with NSCLC

**Fig. 5:**
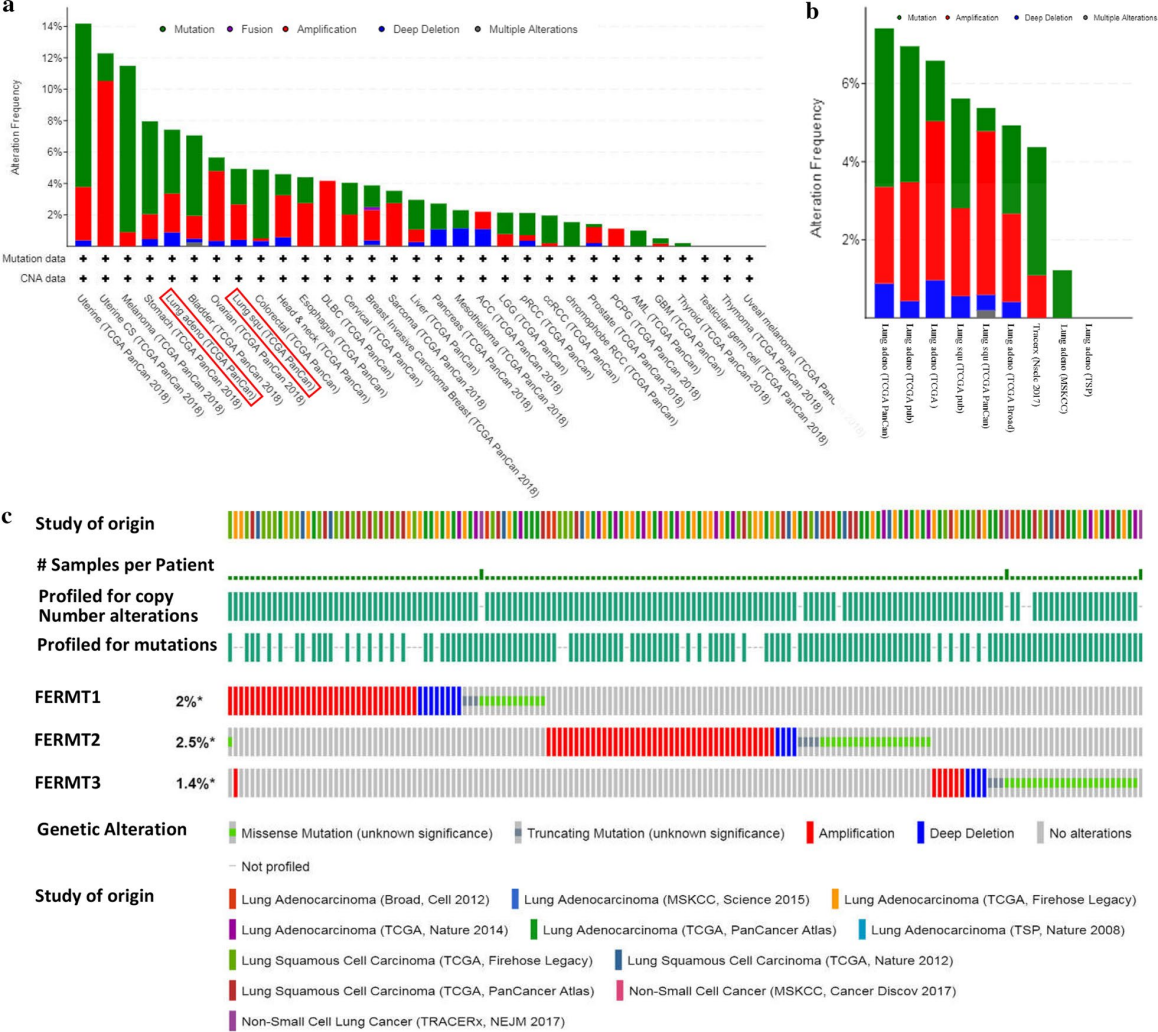
Analysis of Kindlins genetic alterations in NSCLC patients (cBioPortal). **a** Genetic alternations frequencies of Kindlin family members in various carcinoma types. Green, genetic mutations; purple, gene fusions; red, gene amplifications; blue, deep deletions; grey, multiple alterations. **b** Summary of Kindlin family genetic alterations in NSCLC. Alterations frequencies and alterations types of Kindlins were determined in 3897 patients/4181 samples of 11 NSCLC studies. Green, genetic mutations; red, gene amplifications; blue, deep deletions; grey, multiple alterations. **c** OncoPrint visual summary of Kindlins alterations in NSCLC. green, missense mutations. gray, truncation mutation. Red, gene amplifications; blue, deep deletions

We then explored the gene alterations of Kindlins in NSCLC using the cBioPortal database. The Kindlins gene alterations were analyzed in 31 cancer studies, which included 10,931 samples. The results showed that gene alterations in Kindlins were present in different types of NSCLC, including LUSC and LUAD, compared with other cancer types (Fig. 5a). Furthermore, alterations frequencies and alterations types of Kindlins were determined in 3897 patients/ 181 samples of 11 NSCLC studies (Fig. 5b). The results indicated that 5.45% of the 3025 cases of NSCLC had mutations, amplifications, deep deletions and multiple changes in Kindlins, the frequencies were 2.25% (68 cases), 2.64% (80 cases), 0.53% (16 cases) and 0.03% (1 case), respectively, and gene amplifications and mutations accounted for the majority. As shown in Fig. 5c, the percentages of gene alterations in individual Kindlins in NSCLC at a range of 1.4–2.5% (FERMT1, 2%; FERMT2, 2.5%; FERMT3, 1.4%;). In addition, it was found that gene amplifications made up the majority of gene alterations for FERMT1 and FERMT2, whereas gene mutations made up the majority of genetic alterations for FERMT3 (Fig. 5c).

### Correlation between Kindlins expression and immune infiltration in NSCLC

**Fig. 6:**
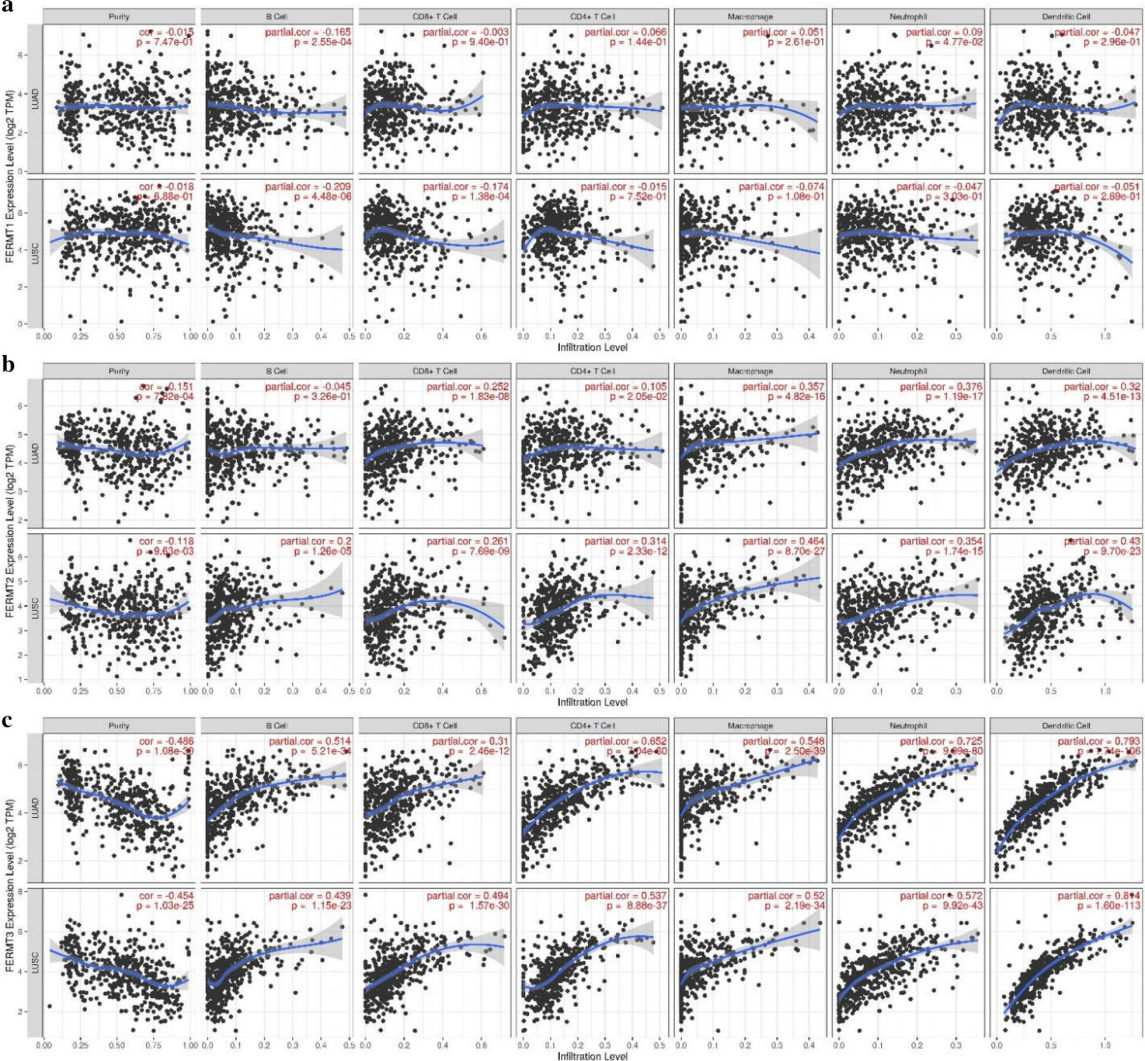
Association of Kindlins (FERMT1, FERMT2, and FERMT3) expression with immune infiltration of immune cells in LUAD and LUSD patients (TIMER). Correlation was analyzed using Pearson’s Correlation

Since immune cells are closely related to tumor proliferation and progression, we analyzed the correlation between kindlin family members and immune infiltration in LUAD and LUSC using TIMER. We found that FERMT3 expression is significantly negatively associated with tumor purity and remarkably positive correlated with infiltrating levels of B cell, CD8+ T cell, CD4+ T cell, Macrophage, Neutrophil, and Dendritic cell in LUAD and LUSC as presented in Fig. 6c. Additionally, FERMT2 expression showed a similar relationship with tumor purity and the infiltration of CD8+ T cell, CD4+ T cell, Macrophage, Neutrophil, and Dendritic cell in LUAD and LUSC (Fig. 6b). However, the correlation strengths between FERMT1 expression and the six types of immune cells were all relatively weaker (Fig. 6a).

### Interaction network of Kindlins at the gene and protein levels

**Fig. 7:**
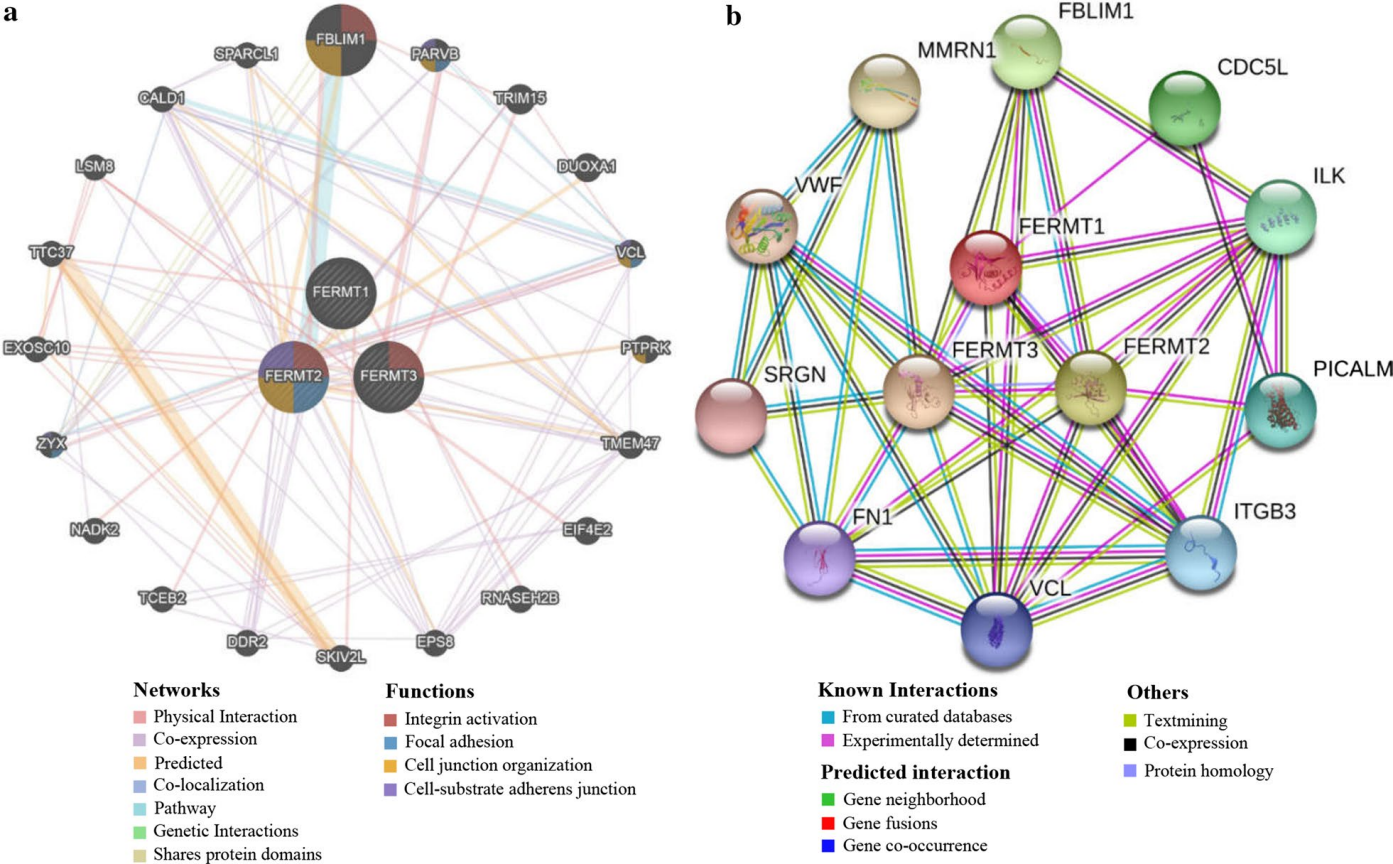
Interaction analysis of Kindlins family members. **a** Gene–gene interaction network of individual Kindlins (GeneMANIA database). **b** Protein–protein interaction network of individual Kindlins (STRING database)

Moreover, the gene–gene interaction network of Kindlins is generated through GeneMANIA. As presented in Fig. 7a, three central nodes implying Kindlins were surrounded by 20 nodes implying genes that were closely associated with Kindlins concerning physical interactions, co-expression, predictions, co-localization, pathway, genetic interactions and shared protein domains. The top five genes showing the greatest connections with the Kindlins contained FBLIM1 (filamin binding LIM protein1), PARVB (Parvin beta), TRIM15 (tripartite motif containing15), DUOXA1 (dual oxidase maturation factor 1) and VCL (vinculin), among which FBLIM1 was linked with FERMT2 for pathway, physical interactions and predictions. Moreover, the functional analysis suggested that these genes were strongly correlated with focal adhesion, integrin activation, cell junction organization and cell-substrate adherens junction.

Using STRING, we also conducted a protein–protein interaction network of the seed genes (FERMT1, FERMT2, and FERMT3) and 20 functional partners with the highest confidence scores (score > 0.900, Fig. 7b). The top five proteins concerning the greatest associations with the Kindlins contained FBLIM1 (filamin binding LIM protein1), CDC5L (cell division cycle 5-like protein), ILK (integrin-linked protein kinase), PICALM (phosphatidylinositol-binding clathrin assembly protein) and ITGB3 (Integrin beta-3). The results indicated that the main biological processes involved the PPI network were platelet degranulation, cell-substrate adhesion, cell adhesion, substrate adhesion-dependent cell spreading, and cell–matrix adhesion, and the main KEGG pathways were focal adhesion, platelet activation, complement and coagulation cascades, amoebiasis and ECM-receptor interaction.

### Functional enrichment analysis of Kindlins in NSCLC

**Fig. 8:**
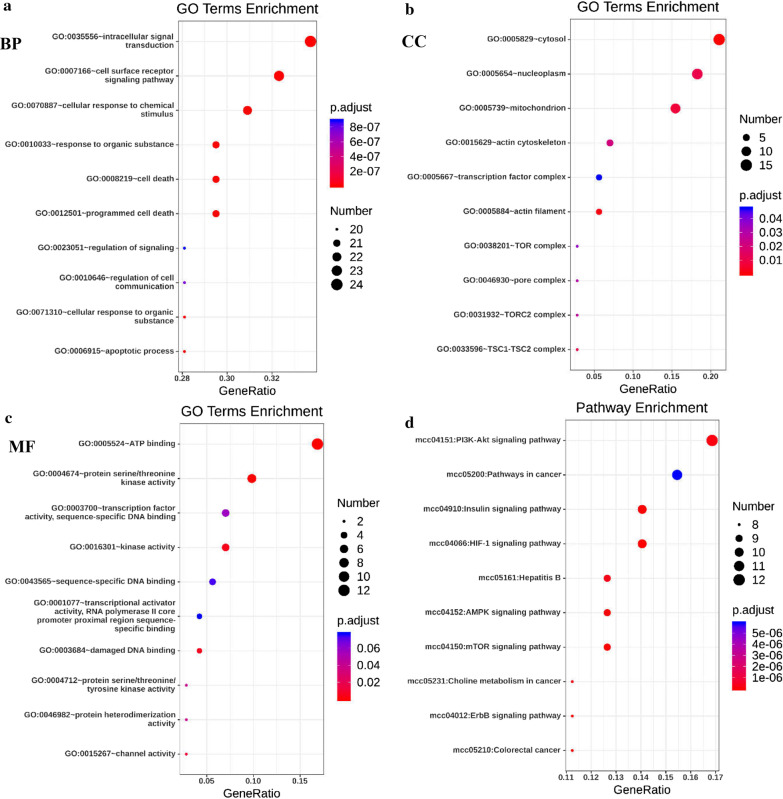
The functional enrichment analysis of Kindlins in Patients with NSCLC (DAVID). Bubble chart of the top 10 results of GO Terms enrichment analysis with biological process (**a**), cellular component (**b**) and molecular function (**c**). (**d**) Bubble chart of the top 10 results of KEGG pathway enrichment analysis. Y-axis: name of the Go Terms function or KEGG pathway; X-axis: percentage of genes assigned to a term in the total number of genes in the network; Bubble size: number of genes assigned to Go Terms function or KEGG pathway. Gene count and *P* values were considered to obtain important metabolic processes

Proteomic data associated with differentially expressed Kindlins in NSCLC were harvested by Reverse Phase Protein Array (RPPA) from cBioportal (S2). These data suggest that *CTLA4, SQSTM1, CDKN1B, GATA3, FASN, PARK7, BAX, RICTOR, NAPSA, PDK1, MACC1, PEA15, BCL2, GSK3A, GSK3B, TSC2, STAT3, AKT1, AKT2, AKT3, TSC1, AXL, PDCD1, MAPK1, MSH2, RPS6KB1, BID, MTOR, EIF4EBP1, BAP1, ETS1, KEAP1, XRCC5, ERBB3, TP63, CASP3, PXN, FOXO3 and NOTCH* (*P* value < 0.05) were primarily correlated with genomic alterations of Kindlins in NSCLC. Furthermore, enrichment analysis of GO terms and KEGG pathways of these differential proteins was performed using DAVID (S3-6). The top 5 results of GO terms analysis with the terms of biological process analysis were intracellular signal transduction, cell surface receptor signaling pathway, cellular response to chemical stimulus, programmed cell death and cell death (Fig. 8a). For cellular components, Kindlins were significantly enriched in the cytosol, nucleoplasm, mitochondrion, actin cytoskeleton and actin filament (Fig. 8b). Molecular function mainly included ATP binding, protein serine/threonine kinase activity, kinase activity, transcription factor activity and sequence-specific DNA binding (Fig. 8c). Moreover, KEGG pathway enrichment analysis showed that, based on gene count and *P* value, the PI3K-Akt signaling pathway is regarded to be the most significant for Kindlins in NSCLC. Additionally, KEGG pathway enrichment included pathways in cancer, HIF-1 signaling pathway, insulin signaling pathway and mTOR signaling pathway (Fig. 8d). To sum up, the above results indicated that Kindlins and their related differential proteins were closely linked to tumor-related signaling pathways in NSCLC.

## Discussion

Kindlins belong to the newly discovered focal adhesion proteins. Previous studies have shown that Kindlins were aberrantly expressed in various human cancer types and these dysregulations have involvement in proliferation, epithelial-mesenchymal transition, migration, invasion, and metastasis [35, 36]. However, the complex roles and significance of Kindlins in NSCLC remain unclear. This study for the first time explored the expression patterns, prognostic value, immune infiltration and potential functions of Kindlins in NSCLC. We found Kindlins could play a tumor-promoting or tumor-suppressive role in NSCLC development. Compared with normal tissue, FERMT1 was highly expressed and correlated a worse prognosis in patients with NSCLC, while FERMT2 had an opposite effect. Besides, the expression level of FERMT3 was reduced in patients with NSCLC and higher FERMT3 mRNA levels had longer OS, although the prognosis was not statistically significant. Additionally, a genetic alteration rate of Kindlins (5.9%) was found in patients with NSCLC. These results indicated that Kindlins play critical roles in NSCLC.

FERMT1, also known as Kindlin-1 and URP1, is primarily expressed in epithelial cells [37]. Prior studies have revealed that FERMT1 is overexpressed and acts as a tumor promoter in various cancer types, including breast [38, 39], colon [40], pancreatic [41], and hepatocellular [42] cancer. Mechanistically, several interesting linkages have indicated that FERMT1 may be involved in tumor occurrence and development by regulating the transforming growth factor-beta (TGFβ) signaling [39, 40, 43]. It was reported that FERMT1 was overexpressed in NSCLC and inhibited tumor epithelial-mesenchymal transition, growth and invasion [12, 13]. Adversely, Sin et al. reported that FERMT1 expression was related to a poor prognosis in lung and breast adenocarcinoma, and FERMT1 may be a strategy for inhibiting metastasis [39]. A similar tumorigenic effect of FERMT1 in NSCLC was also shown in our study. The expression of FERMT1 was remarkably increased in patients with NSCLC and NSCLC cell lines, and FERMT1 was significantly associated with tumor grade and nodal metastasis. Moreover, the higher FERMT1 expression was significantly related to poor OS in NSCLC. These results suggested that FERMT1 might act as a potential therapeutic target in NSCLC.

FERMT2, also known as Kindlin-2 and MIG-2, is widely expressed but not in blood cells [37]. The potential role of FERMT2 in various types of cancer has been extensively explored. It has been suggested that FERMT2 acts either as a tumor promoter or suppressor in different cancers. Recent research has demonstrated that FERMT2 is overexpressed in many tumor types, including gastric [5], breast [8, 9], kidney [44], glioblastoma [45], pancreatic [6, 7], bladder [46], prostate [47], hepatocellular [48] and esophageal [49] cancer, and associated with tumor progression and poor prognosis. In addition, studies have shown that FERMT2 can down-regulate and inhibit tumor cell functions in epithelial ovarian cancer [10], colorectal cancer [11] and mesenchymal cancer cell [50]. In lung cancer, the expression pattern of FERMT2 in previous studies is controversial. Weinstein et al. found that the expression of FERMT2 did not observe a significant change in lung cancer [12]. Zhan et al. reported that FERMT2 was overexpressed in LCC, weakly expressed in LUAD and LUSC and promoted tumor invasion and growth in NSCLC [13]. The present study showed that FERMT2 was markedly decreased in NSCLC tissues and NSCLC cell lines. The previous result [13] was inconsistent with ours, which may be caused by the small sample size and different detection methods in different studies. Additionally, higher FERMT2 expression was strongly associated with long-term OS and FP in NSCLC, and FERMT2 was correlated with tumor grade and nodal metastasis. Therefore, FERMT2 may be used as a prognostic biomarker for NSCLC.

FERMT3, also known as Kindlin-3 and URP2, is mainly expressed in hematopoietic cells [51]. It was reported that FERMT3 was down-regulated and plays a tumor suppressor in many solid tumors including breast cancer, melanoma, as well as lung cancer [14]. Nevertheless, its function as a cancer suppressor or promoter is controversial. Previous research showed that FERMT3 was overexpressed in breast cancer and enhances cancer invasion and metastasis [52]. Moreover, Lu et al. found that FERMT3 was obviously upregulated in glioma, and knockdown of FERMT3 effectively suppressed glioma cell proliferation and chemoresistance [53]. Our study was consistent with the above that FERMT3 expression was significantly decreased in NSCLC, while the expression was not obviously associated with different tumor stages and OS in NSCLC patients. Hence, the prognostic significance of FERMT3 in NSCLC requires further investigation.

The tumor microenvironment (TME) serves an important role in tumor proliferation and progression and is considered as a potential predictor of clinical outcome and immunotherapy reactivity [54]. Our study found that the expression of FERMT2 and FERMT3 was remarkably correlated with the immune infiltration of six immune cells (B cells, CD8+ T cells, CD4+ T cells, macrophages, neutrophils, and dendritic cells) in LUAD and LUSC, suggesting that FERMT2 and FERMT3 may be potential biomarkers of NSCLC for immune checkpoint blockade therapy.

Mechanically, GeneMANIA and STRING database showed that Kindlins and their interacting genes and proteins are strongly correlated with integrin activation, cell junction, focal adhesion and cell adhesion. This suggests that Kindlins may be involved in the tumor progression by regulating integrin activation, cell junction, and focal adhesion. Enrichment analysis indicated that the functions of Kindlins and their related differential proteins are mainly associated with the intracellular signal transduction, cell surface receptor signaling pathway, cellular response to chemical stimulus, programmed cell death and cell death in NSCLC. These findings were consistent with previous studies that Kindlins serve important roles as regulators of integrin inside-out signaling [55]. Furthermore, enrichment analysis suggested that Kindlins were primarily correlated with the PI3K-Akt signaling pathway, pathways in cancer, HIF-1 signaling pathway, insulin signaling pathway and mTOR signaling pathway. These data suggest that Kindlins may modulate tumor development and progression by regulating these signaling pathways in NSCLC, emphasizing their potential as anti-NSCLC therapeutic targets.

There were some limitations to this study that need to be noted. First, our study was primarily based on the transcriptional level, whereas protein expressions need further investigation. Additionally, this study didn't carry out experiments on the biological mechanisms of Kindlins in NSCLC. To address these issues, further in vitro and in vivo studies need to validate these findings.

## Conclusion

In summary, we analyzed the prognostic value and immune infiltration of individual Kindlins in NSCLC through RT-PCR combined with bioinformatics analysis. The expression level of FERMT1 was significantly elevated in NSCLC tissues and NSCLC cell lines, and the expression levels of FERMT2 and FERMT3 were reduced. We also found that FERMT1 expression was significantly associated with short OS in NSCLC patients, while higher FERMT2 expression was strongly associated with better OS and FP. In addition, FERMT2 and FERMT3 were significantly correlated with immune infiltration. Collectively, the research provides a new perspective on the distinct roles of Kindlins in NSCLC and likely has important implications for future effective therapeutic targets in NSCLC.

## Supplementary Information

**Additional file 1**. Table S1: Association of the expression of Kindlins with OS in NSCLC patients with different clinical parameters

**Additional file 2**. Proteomic data

**Additional file 3**. GO Biological Process

**Additional file 4**. GO Cellular Component

**Additional file 5**. GO Molecular Function

**Additional file 6**. KEGG pathway

## Data Availability

All methods were performed in accordance with the relevant guidelines and regulations; Direct web links of datasets about; Oncomine: http://www.oncomine.org; GEPIA database: http://gepia.cancer-pku.cn/; UALCAN: http://ualcan.path.uab.edu; CCLE: www.broadinstitute.org/ccle; Kaplan Meier plotter: http://kmplot.com/analysis; cBioPortal:http://cbioportal.org; TIMER: https://cistrome.shinyapps.io/timer/; GeneMANIA: http://www.genemania.org; STRING: https://string-db.org/; DAVID: https://david.ncifcrf.gov.

## Abbreviations

NSCLC: Non-small cell lung cancer
LUAD: Lung adenocarcinoma
LUSC: Lung squamous cell carcinoma
LCC: Lung large cell carcinoma
GEPIA: Gene expression profiling interactive analysis
cBioPortal: CBio cancer genomics portal
STRING: Search tool for the retrieval of interacting genes
DAVID: The database for annotation visualization and integrated discovery
OS: Overall survival
FP: First progression
PPS: Post-progression survival
PPI: Protein–protein interaction
HR: Hazard ratio
CI: Confidence intervals
