# Supplementary material for: Comprehensive analysis of prognostic value and immune infiltration of kindlin family members in non-small cell lung cancer
Source: BMC Med Genomics. 2021 May 2;14:119. doi: 10.1186/s12920-021-00967-2 (PMC8091749; doi:10.1186/s12920-021-00967-2)
Supplement: Supplementary file 1 — Additional file 1. Table S1: Association of the expression of Kindlins with OS in NSCLC patients with different clinical parameters [file 12920_2021_967_MOESM1_ESM.doc]

**Supplementary Table 1. Association of the expression of Kindlins with OS in NSCLC patients with different clinical parameters.**

| **SUBTYPES** | **FERMT1** | |  | **FERMT2** | |  | **FERMT3** | |
| --- | --- | --- | --- | --- | --- | --- | --- | --- |
|  | **HR (95%CI)** | **P-value** |  | **HR (95%CI)** | **P-value** |  | **HR (95%CI)** | **P-value** |
| **Histology** |  |  |  |  |  |  |  |  |
| LUAD | 1.1 (0.86-1.39) | 0.46 |  | 0.5 (0.39-0.63) | **6.4e-09** |  | 1.1 (0.86-1.39) | 0.46 |
| LUSC | 0.9 (0.66-1.23) | 0.52 |  | 0.94 (0.74-1.19) | 0.6 |  | 0.9 (0.66-1.23) | 0.52 |
| **Stage** |  |  |  |  |  |  |  |  |
| 1 | 0.77 (0.56-1.06) | 0.1 |  | 0.42 (0.32-0.56) | **5.1e-10** |  | 0.77 (0.56-1.06) | 0.1 |
| 2 | 0.82 (0.57-1.19) | 0.29 |  | 0.84 (0.59-1.22) | 0.37 |  | 0.78 (0.49-1.23) | 0.28 |
| 3 | 1.22(0.71-2.1) | 0.47 |  | 1.12 (0.65-1.91) | 0.69 |  | 1.03 (0.51-2.06) | 0.94 |
| 4 | NA |  |  | NA |  |  | NA |  |
| **Gender** |  |  |  |  |  |  |  |  |
| Female | 1.16(0.92-1.46) | 0.22 |  | 0.67 (0.53-0.85) | **0.00074** |  | 0.82 (0.59-1.16) | 0.26 |
| Male | 1.19 (1.02-1.39) | **0.03** |  | 0.81 (0.69-0.95) | **0.0099** |  | 0.87 (0.71-1.07) | 0.18 |
| **Smoking history** |  |  |  |  |  |  |  |  |
| Smoked | 1.18 (0.96-1.45) | 0.11 |  | 0.84 (0.68-1.03) | 0.093 |  | 2.04 (1.34-3.11) | **0.00075** |
| Never smoked | 1 (0.57-1.74) | 1 |  | 0.26 (0.14-0.49) | **8.2e-06** |  | 1.09 (0.49-2.43) | 0.84 |

LUAD, lung adenocarcinoma; LUSC, lung squamous cell carcinoma; Signifcant results are marked in bold; HR, hazard ratio; CI, confdence intervals.
